# Stabilization of Near Identical Hydrogen Bonded Octameric Water Clusters in Crystal Structures of Three Distinct Non-Charged Polyamide Macrocyclic Host Molecules

**DOI:** 10.3390/molecules26092787

**Published:** 2021-05-09

**Authors:** Kajetan Dąbrowa, Magdalena Ceborska, Janusz Jurczak

**Affiliations:** 1 Institute of Organic Chemistry, Polish Academy of Sciences, 01-224 Warsaw, Poland; kdabrowa@icho.edu.pl; 2 Institute of Physical Chemistry, Polish Academy of Sciences, 01-224 Warsaw, Poland; mceborska@ichf.edu.pl

**Keywords:** water clusters, isostructural crystals, macrocyclic compounds, unclosed cryptands

## Abstract

In this paper, we present a comparative analysis of the solid state structures of three well-resolved hydrates of macrocyclic host molecules **1a**, **1b**, and **2** containing an intrannular amide-aryl substituent (lariat arm) connected to a fixed 26-membered ring in a normal (-NHCOAr, hosts **1a** and **1b**) or reverse manner (-CONHAr, host **2**). Despite different chemical structures, these hosts crystallize as isostructural tetrahydrates in the same *P*-1 space group. Moreover, their crystals exhibit identical hydrogen bond motifs resulting in a stabilization of an almost identical unusual octameric water cluster built from the cyclic tetramer core and four water molecules, attached sequentially in an “up-and-down” manner. Further analysis reveals that, among the series, the structure of host **2** provides the most suitable environment for the accommodation of this type of water cluster.

## 1. Introduction

Crystal engineering relies on understanding how multiple weak intermolecular interactions influence the organization and packing of molecules in the solid state, and the application of such knowledge in the development of new solids characterized by specific physical and chemical properties [1]. One of its main tasks is the answer to the problem of what is the dependence between the molecular structure and crystal form. Isostructurality is defined as a phenomenon describing different systems exhibiting similarity in their structures [2]. Isostructurality in single-component organic crystals appears for compounds of closely related molecular structures. There are several reports where the exchange of functional groups does not alter the packing in the crystal structure. Functional groups best known to exhibit such behavior are halogen groups [3]. Additionally, the exchange of Me/Cl [4], Me/Br [5], as well as CH_2_/NH substitution [6], are also well known to result in isostructurality of the structures. Occurrence of 3D isostructurality in the presence of strong hydrogen bonds, namely N-H⋯N with the presence of weak C-H⋯N [7], C-H⋯F, C-H⋯π [8] interactions and π-π stacking was also reported. Bucar et al. [9] showed that isostructural crystals can be obtained by an exchange of a > C=O to > C=S, but only if the studied oxygen/sulfur atom is not involved in hydrogen bonding. Isostructurality induced by a change of the substituent in *para*-position (-NO_2_/-OMe) of a lariat arm in macrocyclic pentaamides was also reported [10]. Isostructurality is more likely to occur for multicomponent crystals [11], where only one of the components is changed. It can be seen for two-component host–guest inclusion compounds [12], as well as for three-component crystals [13]. Isostructurality induced by the solvent is also observed [14,15]. It is a phenomenon often found in pharmaceutical molecular salts [16], and cocrystals [17,18,19]. There are not many examples involving multi-component macrocyclic isostructural crystals, they include solvates of tetrahaloethynyl cavitands [20] and complexes of cucurbit [6] uril with three glycine containing dipeptides [21].

Unclosed cryptands (UCs) [22,23,24,25,26,27] belong to the class of fairly new macrocyclic receptors, structurally related to well-established cryptands [28,29,30,31,32]. They are, however, much more flexible than their rigid analogs, as they are built from the macroring of various sizes to which is connected a labile intrannular substituent–lariat arm. Due to their inherent flexibility, they are able to adjust their shape to highly demanding guests, in particular anions. Lately, we showed that 26-membered pentamide unclosed cryptands are able to accommodate octameric water clusters in their structure [10,33], proving that such molecules are a good choice for studying the hydration phenomena of small organic compounds, as well as serving as a model for more complicated structures of biological importance. It may prove very useful as studying the hydration behavior of biological molecules still presents a challenge for modern science [34,35].

We are focused on investigating the crystal structures of closely related macrocyclic compounds, which allows us to examine how incremental structural changes affect the crystal packing [10,36,37]. During our study, we proved that the presence of *para*-substituent in the lariat arm of 26-membered unclosed cryptand is essential for the stabilization of octameric water cluster [10]. It is worth mentioning that such stabilization occurs for UCs possessing amido-phenyl group in lariat arm substituted with electron-withdrawing (-NO_2_, as in host **1a**), as well as electron-donating group (-OMe, as in host **1b**). On the contrary, structural studies of a macrocycle equipped with an unsubstituted amido-phenyl substituent revealed the occurrence of a water chain stabilized by two unclosed cryptand molecules.

**Figure 1:**
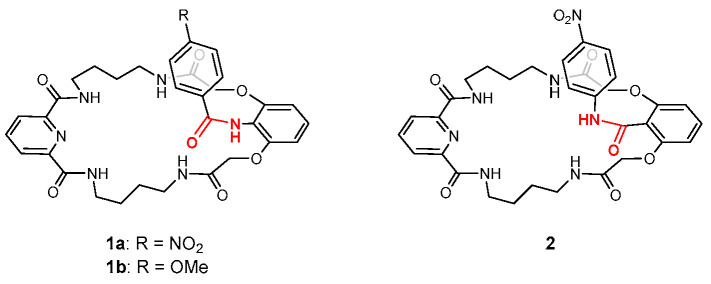
Molecular structures of structurally related macrocyclic host-guest systems **1** and **2**.

In the present paper, we investigate how the attachment of an intraannular amido-*p*-nitrophenyl substituent (lariat arm) on the 26-membered unclosed cryptand of type **1** influences the molecular packing and possible stabilization of water clusters in so-designed host **2** (see the red-colored amide groups in Figure 1).

Previous solution studies reveal that such structural isomerism of the amide group has a beneficial impact on the molecular recognition of anions [22,24]. The host **2** has been synthetized and its crystal structure was determined by X-ray crystallography. It was found, unexpectedly, that host **2** contains near the same octameric water cluster as previously found in the crystal structures of analogs **1a [33]** and **1b [10]**. A comparative crystal structure analysis has been carried out to identify the role of macrocyclic skeleton and lariat arm on the formation of water clusters in the crystal lattice of hosts **1a**–**b** and **2**.

## 2. Results and Discussion

**Scheme 1:**
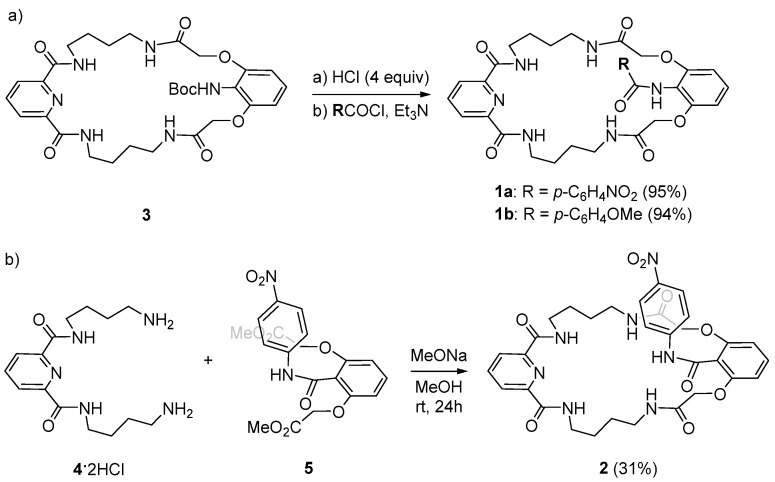
Synthetic route towards the studied macrocyclic hosts (**a**) **1a**, **1b,** and (**b**) **2**.

The target macrocyclic hosts **1a** and **1b** were obtained in very high yields using a “one-pot” post-macrocyclisation procedure involving deprotection and functionalization of Boc-protected macrocyclic amine **3** (Scheme 1a) [27], whereas the novel host **2** was prepared by a direct MeONa-assisted macrocyclization [10] between α,ω-diester **4** and hydrochloric salt of α,ω-diamine **5** (Scheme 1b).

**Figure 2:**
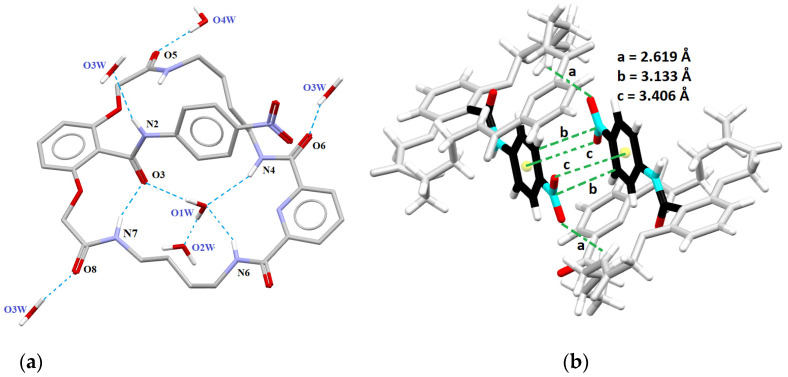
X-ray structure of **2**∙(H_2_O)_4_ showing intermolecular H-bonds (**a**) and short contacts (**b**).

Similar to the previously described unclosed cryptands **1a** and **1b,** macrocycle **2** crystallizes as a pair of enantiomers in a triclinic space group *P*-1. In the solid state, unclosed cryptands in general may adopt three different conformations: *T-*, *C-*, and *S*-shaped [37]. All three macrocycles studied here, **1a, 1b,** and **2,** adopt *C*-shaped conformation. The *C*-shaped conformation of macrocycle **2** is stabilized by a net of 8 inter- and intramolecular hydrogen bonds (Figure 2a).

Molecules of **2** form dimers, where nitro-substituted aromatic rings from the lariat arms of adjacent host molecules are arranged in an antiparallel mode and are connected via CH⋯O, as well as π-stacking interactions (Figure 2b). In addition, the lariat arm is held in its position via H-bond between N7 nitrogen atom of the main macroring and the carbonyl atom O3 in the lariat arm, which is further connected with O1W molecule. The further stabilization is realized through interactions with water molecules O2W, O3W, and O4W (for details describing hydrogen bonds, see Table 1).

### 2.1. Stabilization of Discrete Octameric Water Cluster

**Figure 3:**
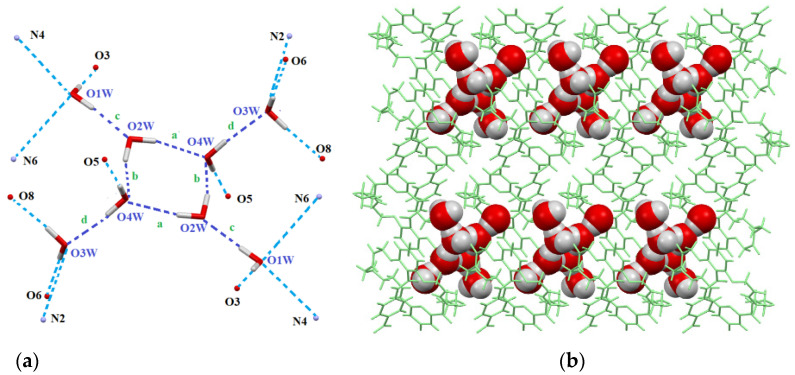
Hydrogen bonds in water octamer stabilized in the structure of **2** (**a**) and molecular packing of water clusters (**b**).

Further crystal analysis reveals that the confined space of unclosed cryptand **2**, similarly to the previous cases of hosts **1a** and **1b**, stabilizes a discrete water octamer built from a tetrameric cyclic water cluster (for selected examples of discrete water tetramers, see references: [38,39,40,41,42]) with four water molecules attached in an “up-and-down” manner to each corner of the tetramer (O1W, O2W, O3W, and O4W; Figure 3).

The average distance between water molecules in cyclic tetramer (O2W-O4W) equals to 2.853 Å, while average distance between water molecules from the tetramer and dangling water molecule O1W and O3W equals to 2.768 Å, which gives an overall average of 2.811 Å (all H-bonds in the octameric water cluster are summarized in Table 1, entries 10–13). The core cyclic water tetramer is higher in energy by at least 35.3 kcal·mol^−1^ than the global minimum with the cage symmetry [33,43]. This indicates a large energetic compensation of this water assembly by the crystal lattice. 

### 2.2. Comparative Analysis of Crystals

To elucidate the role of a macrocyclic skeleton and the lariat arm on the formation and stability of the discrete water cluster, we conducted a comparative crystal structure analysis of tetrahydrates of hosts **1a**–**b** and **2**. The three studied compounds (**1a**, **1b**, and **2**) crystallize in the triclinic space group *P*-1 and their unit cell parameters and cell volumes exhibit very similar values (Table 2).

**Figure 4:**
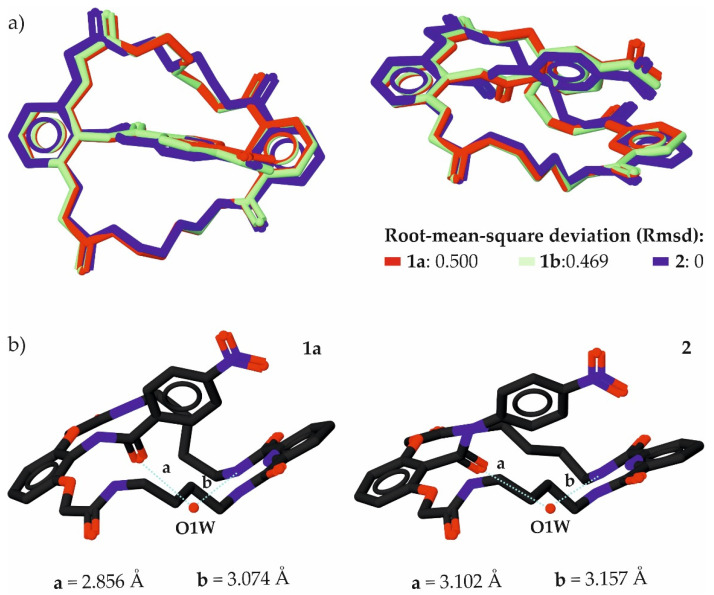
(**a**) Superposition of structures of macrocyclic host molecules: **1a** (red), **1b** (light green), and **2** (blue) and (**b**) stabilization of the macrocycle conformation via H-bonds with O1W-**1a** (left) and **2** (right). Hydrogen atoms were omitted for clarity.

In addition to having very similar cell parameters, the crystal structures of hosts **1a**, **1b**, and **2** also display similar crystal packing features. All hosts crystallize as a pair of enantiomers and adopt *C*-shaped conformation (the overlay of three molecules of unclosed cryptands **1a**, **1b**, and, **2** is presented in Figure 4a). It can be seen from the superposition of **1a**, **1b**, and **2** that, although the lariat arms of **1a** and **1b**, which differ in the *p*-substituent in the terminal phenyl group overlay perfectly, the lariat arm of **2** is slightly shifted. It is due to the reversed position of the amide group in the lariat arm which bonds with O1W molecule via O-H⋯O interactions (Figure 4b).

**Figure 5:**
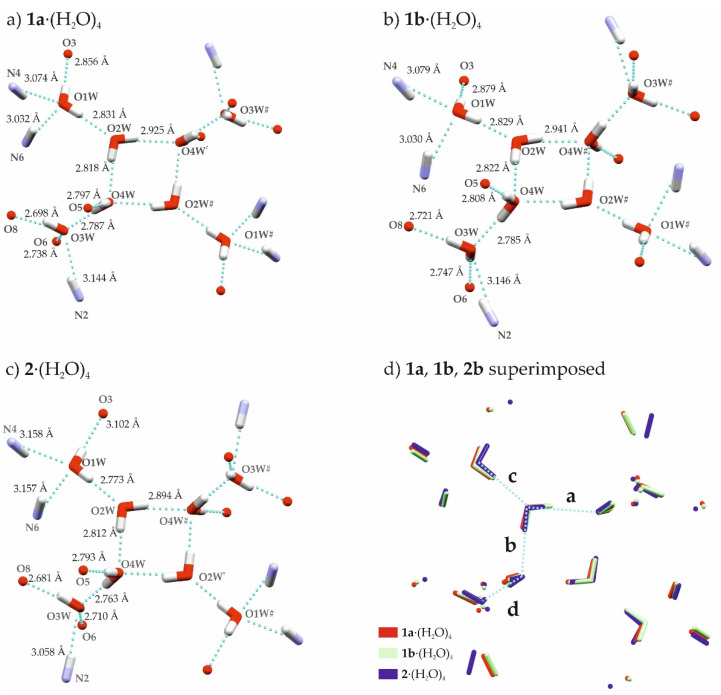
Comparison of hydrogen bonds in water octamers in structures of hosts **1a** (**a**), **1b** (**b**), and **2** (**c**), and overlay of clusters in **1a** (red), **1b** (light green), and **2** (blue) (**d**); labels with “#” denote symmetry equivalent atoms.

Despite the obvious differences in the structures of unclosed cryptands **1a**, **1b**, and **2**, all of them exhibit very similar hydrogen bond motifs, which results in a stabilization of similar octameric water clusters (Figure 5, Table 3 and Table S1).

The average distance between the oxygen atoms of water molecules forming water cluster is 2.841 Å, 2.925 Å, and 2.811 Å in the crystal structures of hosts of **1a**, **1b**, and **2**, respectively. This parameter is related to the relative stability of the water assembly in a confined environment. It can be therefore concluded that, among the studied macrocyclic compounds, host **2** provides the most suitable molecular architecture for the formation of this type of water cluster.

**Figure 6:**
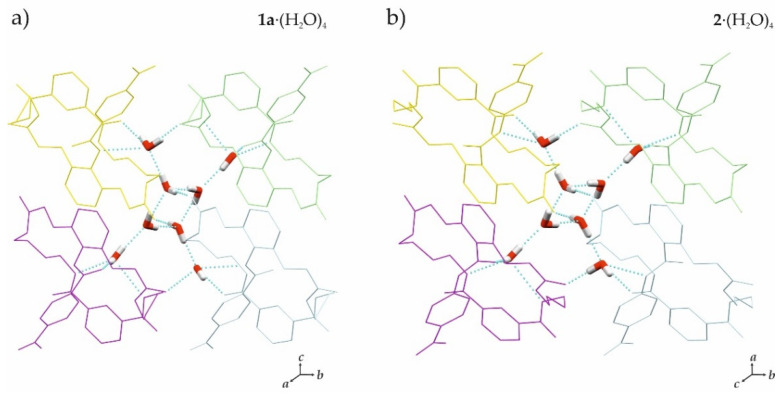
Crystal structures of water tetramers of isomeric macrocyclic hosts **1a** (**a**) and **2** (**b**) exemplifying the similar molecular environment around octameric water clusters (shown in capped stick representation); nonrelevant protons were omitted for clarity.

**Figure 7:**
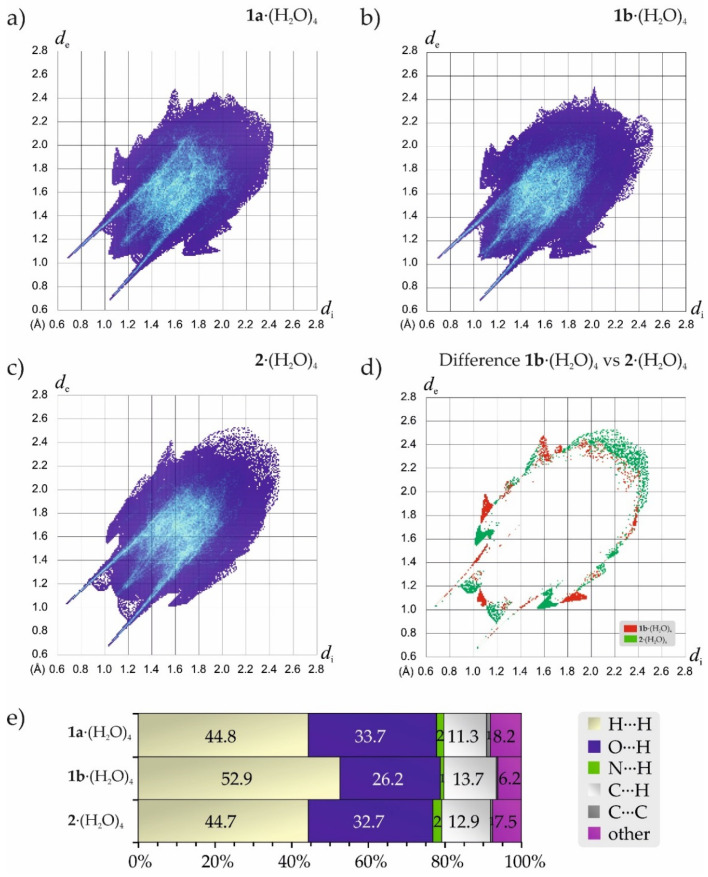
Hirschfeld fingerprint plots for crystals of **1a**·4H_2_O (**a**), **1b**·4H_2_O (**b**), **2**·4H_2_O (**c**), difference between the corresponding fingerprint plots of tetrahydrates of **1a** (colored in red) vs. **2b** (colored in green) (**d**), and percentage contributions to the Hirschfeld surface area for the various close intermolecular contacts (**e**, from top-left to bottom-right)**.**

To get a better insight into the role of the attachment of lariat’ amide group (i.e., “normal” vs. “reverse” manner), we further closely inspected the molecular environment around the water cluster, as well as the geometrical parameters describing the water cluster, with particular emphasis on closely related isomeric hosts **1a** and **2** (Figure 6 and Figure 7, Table S1).

At first sight, the analysis of the molecular environment reveals a close similarity between the crystal structures of hosts **1a** and **2**, however, closer inspection reveals some important differences, in particular in the arrangements of host molecules and the conformation of aliphatic butylene linkers. These differences are confirmed by the Hirshfeld fingerprint plots (Figure 7a,c,d) and a very comparable share of intermolecular interactions (Figure 7e).

The weak and nondirectional van der Waals interactions account for nearly 56% of the total surface area, in which H⋯H dominates over C–H⋯C *π* contacts (45% vs. 11–13%). The short and highly directional hydrogen bonds (mainly between water molecules and with water molecules hydrogen bonded to oxygen carbonyl atoms and NH amide protons) account for 33–34% of the total surface area.

In contrast, the share of intermolecular interactions for hosts **1a** and **2** vs. **1b** highlights much larger differences, plausibly related to the presence of different *para*-substituent (-NO_2_ for **1a** and **2** vs. -OMe for **1b**). Although all these three hosts **1a**, **1b**, and **2** crystallize as isostructural tetrahydrates, it is evident that replacement of the *para*-substituent from the nitro- to methoxy group markedly increases the share of H⋯H and C–H⋯H interactions (56.1%, 57.6% vs. 66.6% for **1a** and **2** vs **1b**, respectively), whereas the share of O⋯H interactions becomes smaller (33.7%, 32.7% vs. 26.2% for **1a** and **2** vs. **1b**, respectively).

More importantly, analysis of the lengths and angles of molecular interactions in the water clusters reveals that water cluster is more stable in the crystal lattice of host **2** than in host **1a**. In particular, the hydrogen bonds are considerably shorter (up to 0.06 Å), as well as the angles between water molecules are more directional (e.g., angle (c) is 171.6° vs. 176.6° in **2** and **1a**, respectively). This clearly highlights that the molecular environment of host **2** is much more suitable for the formation of confined water octamer.

## 3. Materials and Methods

### 3.1. Reagents and General Methods

All reagents were used as received. The solvents were dried by distillation over the appropriate drying agents. All solvents were obtained from common suppliers and used as received. TLC was carried out on Merck Kieselgel F254 plates (Merck, Germany). Melting points were determined using a hot-stage apparatus and were uncorrected. The NMR spectra were recorded on a Varian Mercury 600 (Varian, Palo Alto, CA, USA) instrument. Chemical shifts are reported in ppm (δ) and are set to the solvent residue peak. *J* coupling constants values are reported in Hz. Mass spectral analyses were performed with the ESI-TOF technique on a Mariner mass spectrometer from PerSeptive Biosystem (Waltham, MA, USA).

Compounds **1a**, **1b**, **4**, and **5** were prepared according to the procedures reported previously [27].

### 3.2. Synthesis of Molecular Host 2

To a stirred solution of diamine dihydrochloride **4** (380 mg, 1.0 mmol) and diester **5** (420 mg, 1.0 mmol) and in anhydrous methanol (100 mL, c = 0.01 M), a freshly prepared solution of sodium methoxide (6.0 mmol, 6 equivalents) in anhydrous methanol (5 mL) was added. The resulting mixture was stirred at rt for 7 days and then silica gel (~3 g) was added. Solvent was evaporated yielding a yellowish residue which was purified by column chromatography using a gradient of methanol in CH_2_Cl_2_ [99:1→95:5 *v*/*v*] as an eluent. Evaporation of the solvent and recrystallization from MeOH yielded the target product **2** (210 mg, 32%) in the form of yellow crystals (mp 169–171 °C). ^1^H-NMR (600 MHz, DMSO-*d*_6_) δ 11.25 (s, 1H), 9.44 (t, *J* 6.2, 2H), 8.25–8.18 (m, 3H), 7.81 (d, *J* 9.2, 2H), 7.61 (t, *J* 5.7, 2H), 7.41 (t, *J* 8.4, 1H), 6.86 (d, *J* 8.5, 2H), 4.62 (s, 4H), 3.28 (dd, *J* 13.7, 6.7, 4H), 3.16 (dd, *J* 11.4, 5.7, 4H), 1.48–1.35 (m, 8H). ^13^C-NMR (150 MHz, DMSO-d6) δ 167.3, 164.5, 162.9, 155.5, 148.8, 144.7, 142.3, 139.3, 132.0, 124.8, 124.2, 118.9, 115.6, 106.7, 67.8, 38.9, 38.1, 26.9, 26.6. HR-MS (ESI) (*m*/*z*) calc. for C_32_H_35_N_7_O_9_Na [M + Na]^+^ 684.23885, found: 684.24116. Anal (%) calc. for C_32_H_35_N_7_O_9_·H2O: C 56.55, H 5.49, N 14.42, found: C 56.40, H 5.37, N 14.57.

### 3.3. Crystallography

The measurement of 2 crystals was performed on a KM4CCD κ-axis diffractometer with graphite-monochromated MoK_α_ radiation. The crystal was positioned at 50 mm from the CCD camera. A total of 637 frames were measured at 1° intervals with a counting time of 20 s. The data were corrected for Lorentz and polarization effects. Multi-scan absorption correction has been applied. Data reduction and analysis were carried out with the Agilent program [44]. The structure was solved by direct methods and refined using SHELXL-2014/7 [45], Olex2 v.1.2.10 [46], and WinGX Program System v. 2014.1 [47]. Refinement was based on F^2^ for all reflections except those with very negative F^2^. Weighted *R* factors ω*R* and all goodness-of-fit S values are based on F^2^. Conventional *R* factors are based on F with F set to zero for negative F^2^. The F_o_^2^ > 2σ(F_o_^2^) criterion was used only for calculating *R* factors and is not relevant to the choice of reflections for the refinement. The *R* factors based on F^2^ are about twice as large as those based on F. Most of the hydrogen atoms were located geometrically and their positions–except those engaged in hydrogen bonds-and temperature factors were not refined. Scattering factors were taken from Tables 4.2.6.8 and 6.1.1.4 from the International Crystallographic Tables Vol. C [48]. The graphics was prepared using Mercury 3.9 [49]. The crystal packing coefficients (CP_k_), defined as: CP_k_ = Z × V_mol_ × V_cell_^−1^, where Z is the number of molecules in the unit cell, V_cell_ is the volume of the cell taken from the X-ray structure, and V_mol_ is the volume of geometry-optimized molecule (only hydrogen positions were refined) calculated at DFT/B3LYP-D3/6-31G(d) level of theory using Spartan’18 program [50]. Hirshfeld calculations were performed using the default parameters of the Crystal Explorer 17.5 program [51,52,53,54].

Single monocrystals of compound **2** suitable for X-ray crystallographic analysis were obtained by diffusion of water vapor into DMSO solution of a mixture of **2** and an excess amount of TBAH_2_PO_4_ salt (~10 equivalents). Crystal data for **2**∙(H_2_O)_4_: C_33_H_46_N_6_O_12_, M_r_ = 718.76, colorless plate, triclinic, spacegroup *P*-1, *a* = 10.1059(3) Å, *b* = 10.7645(3) Å, *c* = 16.9216(5) Å, α = 92.533(2)°, β = 104.115(2)°, γ = 101.344(2)°, *V* = 1742.12(9) Å^3^, *Z* = 2, Dc = 1.399 cm^−1^, μ = 0.111 mm^−1^, 37,615 reflections measured, 8195 unique, 521 parameters, *R* = 0.037, ω*R* = 0.081 (*R* = 0.055, ω*R* = 0.09 for all data), Goof = 1.02. CCDC 2062460.

## 4. Conclusions

In conclusion, we present a comparative analysis of the single-crystal X-ray structures of three hydrates of macrocyclic host molecules **1a**, **1b**, and **2** bearing various intrannular substituent (lariat arm) connected to a fixed 26-membered ring in a normal (-NHCO-*p*-C_6_H_4_X, **1a**: X = NO_2_, **1b**: X = OMe) or reverse manner (**2**, -CONH-*p*-C_6_H_4_NO_2_) fashion. Despite structural differences, all hosts crystallize in *P*-1 space group as isostructural tetrahydrates crystals containing an unusual and energetically disfavored [24] octameric water cluster. The water assembly is built from the cyclic tetramer and four dangling water molecules that are placed in alternate “up-and-down” positions. Further examination of crystals, reveals that reversing the mode of attachment of amide group in **2** (relative to compounds **1a** and **1b**), changing the binding mode of relocation induces the conformation of the molecule by the formation of hydrogen bonds via the carbonyl O3 atom (N7-H7⋯O3 and O1W-H1V⋯O3) and amidic N2 atoms (N2-H2⋯O3W). Lariat arms of hosts **1a** and **1b** overlay perfectly, while the lariat arm of host **2,** due to the reversed position of amide group is slightly shifted. This structural difference, however, does not influence significantly the general conformation of the macrocyclic skeleton nor its ability to stabilize water cluster. In fact, this inversion was found to markedly increase the stability of water cluster by host **2** (avg. *d*_O⋯O_ = 2.811 Å) compared to corresponding water assemblies in crystal structures of hosts **1a** (avg. *d*_O⋯O_ = 2.841 Å) and **1b** (avg. *d*_O⋯O_ = 2.925 Å).

## Figures and Tables

**Table 1:** Analysis of hydrogen bonds stabilizing one molecule of host **2** (entries 1–9) and analysis of hydrogen bonds stabilizing water octamer in the structure of **2** (entries 10–13).

Entry	Interaction	*d*(D⋯A) [Å]	*d*(H⋯A) [Å]	<(D-H⋯A) [^o^]
1	N2-H2⋯O3W ^[a]^	3.058	2.214	165.4
2	N4-H4⋯O1W	3.158	2.336	155.0
3	N6-H6⋯O1W	3.157	2.339	156.3
4	N7-H7⋯O3	3.240	2.482	148.3
5	O1W-H1V⋯O3	3.102	2.212	176.2
6	O1W-H1V⋯O2W	2.773	1.924	176.7
7	O3W ^[b]^-H3V ^[b]^⋯O6	2.710	1.884	159.3
8	O3W ^[c]^-H3W ^[c]^⋯O8	2.681	1.831	169.0
9	O4W ^[d]^-H4W ^[d]^⋯O5	2.793	1.940	178.2
10	O2W-H⋯O4W	2.894	2.006	167.69
11	O4W⋯H-O2W’	2.812	1.921	172.68
12	O2W⋯H-O1W	2.773	1.924	176.71
13	O4W-H⋯O3W	2.763	1.881	176.39

^[a–d]^ Symmetry equivalent positions: [a] 1 + x, 1 + y, z; [b] x, 1 + y, z; [c] 1 + x, y, z; [d] 1 − x, 2 − y, 2 - z.

**Table 2:** Comparison of the crystal data of macrocyclic hosts **1a**, **1b**, and **2**.

Entry	Parameter ^[a]^	1a	1b	2	Avg ^[b]^
1	SG	*P*-1 (Triclinic)			-
2	a [Å]	9.6293(4)	9.7432(8)	10.1059(3)	0.1865
3	b [Å]	10.5860(4)	10.5621(9)	10.7645(3)	0.0846
4	c [Å]	18.1620(7)	17.9336(15)	16.9216(5)	0.5005
5	α [^o^]	100.749(3)	95.803(3)	92.533(2)	6.630
6	β [^o^]	105.086(3)	104.647(3)	104.115(2)	13.096
7	γ [^o^]	99.485(3)	99.899(3)	101.344(2)	8.9362
8	Z	2			-
9	Dx [g cm^−3^]	1.398	1.373	1.399	0.011
10	V [Å^3^]	1742.95	1738.61	1743.38	2.02
11	CP^k [b]^	0.792 [33]	0.813	0.805	0.803

^[a]^ SG—space group; a, b, and c—lattice constants; α—angle between b and c; β—angle between a and c; γ—angle between a and b; Z—number of molecules in unit cell; Dx—crystal density, V—volume of unit cell; CPk—crystal packing coefficient, see Section 3.3. for details. ^[b]^ Calculated average of absolute deviations from the mean.

**Table 3:** Selected geometrical parameters describing water clusters stabilized in the structures of isomeric macrocyclic hosts **1a** and **2** ^[a]^.

z	Parameter	1a	2	Diff. ^[b]^
1	a [Å]	2.925	2.894	−0.031
2	b [Å]	2.818	2.812	−0.006
3	c [Å]	2.831	2.773	−0.058
4	d [Å]	2.788	2.763	−0.025
5	<D-H⋯A (a) [^o^]	167.73	167.69	−0.04
6	<D-H⋯A (b) [^o^]	171.4	172.68	1.28
7	<D-H⋯A (c) [^o^]	171.64	176.61	4.97
8	<D-H⋯A (d) [^o^]	172.47	176.39	3.92

^[a]^ For host **1b** see Table S1. ^[b]^ Difference between parameter from the X-ray structure of host **2** and host **1**.

## Data Availability

The data presented in this study are available in article and supplementary information.

## Supplementary Materials

The following are available online, Table S1: Selected geometrical parameters describing water clusters stabilized in the structures of macrocyclic host molecules **1a**, **1b**, and **2**; Figure S1: ^1^H NMR (600 MHz) and ^13^C NMR (150 MHz) spectra of macrocyclic host **2** in DMSO-*d*_6_; Figure S2: 2D HSQC spectrum of compound **2** in DMSO-*d*_6_.

## Sample Availability

Samples of the compounds **1a**, **1b**, and **2** are available in limited quantity from the authors.
