# Supplementary material for: Stabilization of Near Identical Hydrogen Bonded Octameric Water Clusters in Crystal Structures of Three Distinct Non-Charged Polyamide Macrocyclic Host Molecules
Source: Molecules. 2021 May 9;26(9):2787. doi: 10.3390/molecules26092787 (PMC8125911; doi:10.3390/molecules26092787)
Supplement: Supplementary file 1 [file molecules-26-02787-s001.zip › Supplementary_information_file.pdf]

Supporting Information

Stabilization of Near Identical Octameric Water Clusters in Crystal Structures of Three Distinct Non-Charged Macrocyclic Host Molecules

Kajetan Dąbrowa [1], Magdalena Ceborska[2] and Janusz Jurczak[1,*]

[1] Institute of Organic Chemistry,Polish Academy of Sciences, Warsaw, Poland.

[2] Institute of Physical Chemistry, Polish Academy of Sciences, Warsaw, Poland.

* Corresponding author email: jurczak_group@icho.edu.pl;

Contents

1. ADDITIONAL CRYSTAL DATA **2**

2. COPIES OF ^{1}H AND ^{13}C NMR SPECTRA **3**

1. Additional Crystal Data

Table S1. Selected geometrical parameters describing water clusters stabilized in the structures of macrocyclic host molecules **1a**, **1b**, and **2**.

Entry	Parameter	1a	1b	2	Difference 1a vs 2
1	a [Å]	2.925	3.144	2.894	-0.031
2	b [Å]	2.818	2.941	2.812	-0.006
3	c [Å]	2.831	2.829	2.773	-0.058
4	d [Å]	2.788	2.785	2.763	-0.025
5	< D-H···A (a) [°]	167.73	174.31	167.69	-0.04
6	< D-H···A (b)[°]	171.4	166.76	172.68	1.28
7	< D-H···A (c) [°]	171.64	173.25	176.61	4.97
8	< D-H···A (d) [°]	172.47	173.20	176.39	3.92

2. Copies of ^{1}H and ^{13}C NMR Spectra

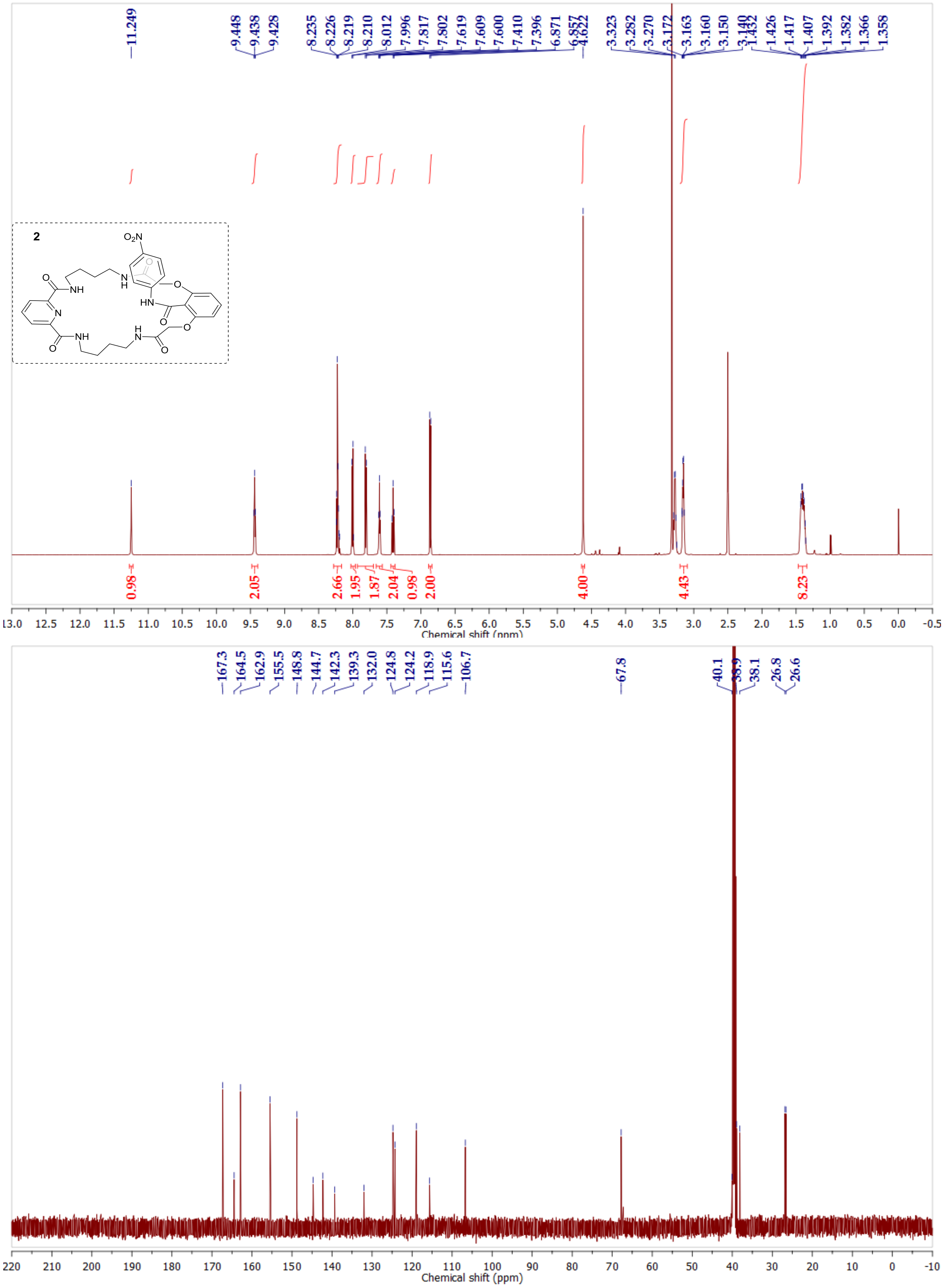

Figure S1. ^{1}H NMR (600 MHz) and ^{13}C NMR (150 MHz) spectra of macrocyclic host **2** in DMSO-d_6.

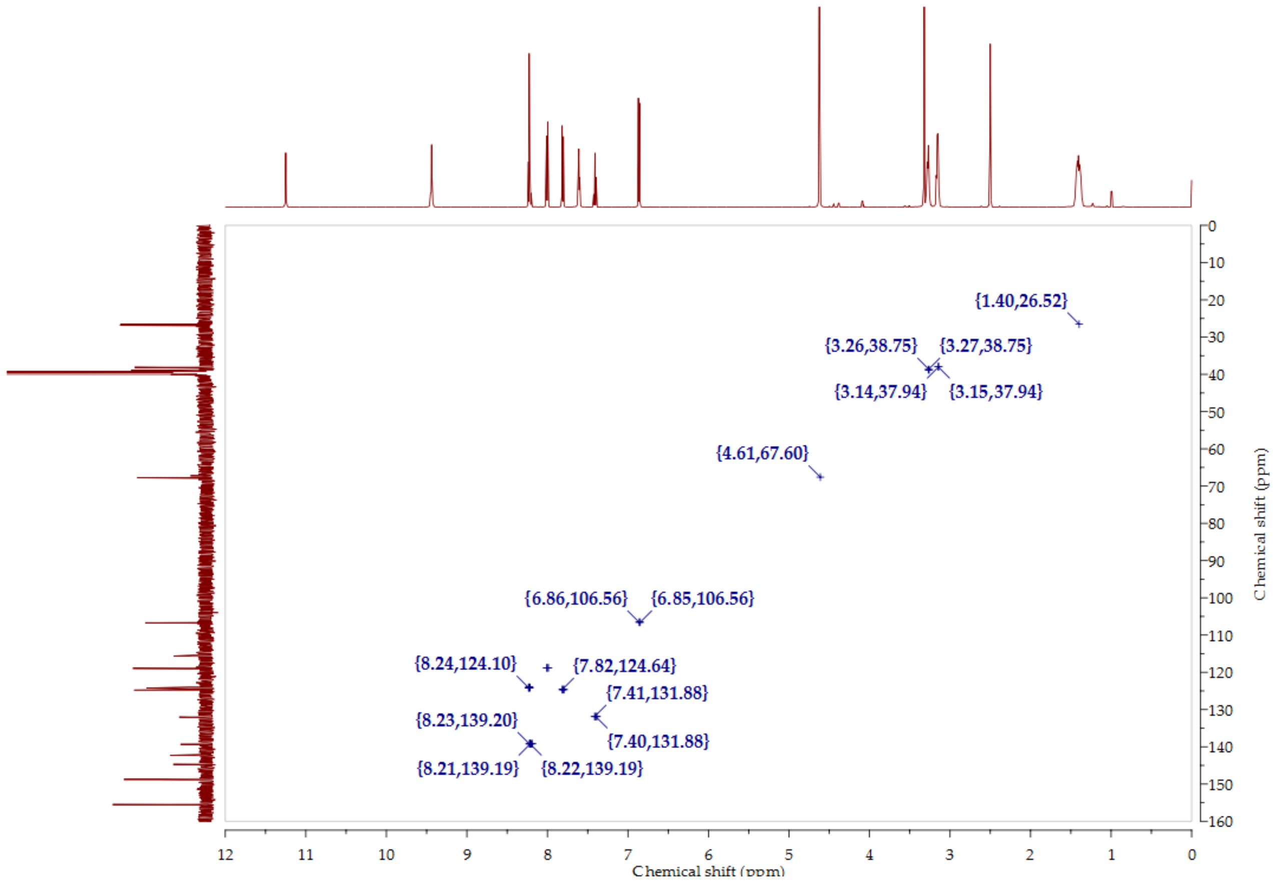

Figure S2. 2D HSQC spectrum of compound **2** in DMSO-d_6.
